# Polygenic risk and causal inference of psychiatric comorbidity in inflammatory bowel disease among patients with European ancestry

**DOI:** 10.1186/s12967-022-03242-9

**Published:** 2022-01-27

**Authors:** Yao Li, Charles N. Bernstein, Wei Xu, Pingzhao Hu

**Affiliations:** 1 Department of Biochemistry and Medical Genetics, University of Manitoba, Room 308-Basic Medical Sciences Building, 745 Bannatyne Avenue, Winnipeg, MB R3E 0J9 Canada; 2 Dalla Lana School of Public Health, University of Toronto, Toronto, ON M5T 3M7 Canada; 3 Department of Internal Medicine and The University of Manitoba IBD Clinical and Research Centre, University of Manitoba, Winnipeg, MB Canada; 4 Biostatistics Department, Princess Margaret Cancer Centre, Toronto, ON M5G 2M9 Canada; 5 CancerCare Manitoba Research Institute, Winnipeg, MB R3E 0V9 Canada

**Keywords:** Polygenetic risk score, Mediation causal inference, Psychiatric comorbidity, Inflammatory bowel disease, Transcriptome-wide association analysis, Genome-wide association analysis, European ancestry

## Abstract

**Background:**

Approximately 40% of persons with inflammatory bowel disease (IBD) experience psychiatric comorbidities (PC). Previous studies demonstrated the polygenetic effect on both IBD and PC. In this study, we evaluated the contribution of genetic variants to PC among the IBD population. Additionally, we evaluated whether this effect is mediated by the expression level of the *RBPMS* gene, which was identified in our previous studies as a potential risk factor of PC in persons with IBD.

**Materials and methods:**

The polygenic risk score (PRS) was estimated among persons with IBD of European ancestry (n = 240) from the Manitoba IBD Cohort Study by using external genome-wide association studies (GWAS). The association and prediction performance were examined between the estimated PRS and PC status among persons with IBD. Finally, regression-based models were applied to explore whether the imputed expression level of the *RBPMS* gene is a mediator between estimated PRS and PC status in IBD.

**Results:**

The estimated PRS had a significantly positive association with PC status (for the highest effect: P-value threshold = 5 × 10^–3^, odds ratio = 2.0, P-value = 1.5 × 10^–5^). Around 13% of the causal effect between the PRS and PC status in IBD was mediated by the expression level of the *RBPMS* gene. The area under the curve of the PRS-based PC prediction model is around 0.7 at the threshold of 5 × 10^–4^.

**Conclusion:**

PC status in IBD depends on genetic influences among persons with European ancestry. The PRS could potentially be applied to PC risk screening to identify persons with IBD at a high risk of PC. Around 13% of this genetic influence could be explained by the expression level of the *RBPMS* gene.

**Supplementary Information:**

The online version contains supplementary material available at 10.1186/s12967-022-03242-9.

## Introduction

Inflammatory bowel disease (IBD) is a chronic immunologically mediated disease affecting the gastrointestinal tract which is manifested by diarrhea, rectal bleeding, and abdominal pain. IBD can affect people of all ages and the incidence of IBD peaks in young adulthood [1]. Although IBD can be observed across the world, Canada is among the countries with the highest disease prevalence. Nearly 300,000 Canadians have been diagnosed with IBD [2, 3].

Approximately 40% of persons with IBD experience psychiatric comorbidity (PC), such as depression and anxiety [4], which is significantly higher than in the general population [5]. Comorbid psychiatric illness could lead to a higher risk of disease relapse and poorer response to treatment [6–8]. However, the impact of PC could be controlled by early intervention with relevant psychotherapies [9, 10]. Therefore, identifying IBD patients with a higher risk of PC could improve the long-term treatment outcome.

Both IBD and psychiatric disorders have complex etiology involving interactions between genetic and environmental factors [11–13] and are known to be highly polygenic [14, 15]. In IBD, over 200 single nucleotide polymorphisms (SNPs) have been identified as risk loci for IBD by genome-wide association studies (GWAS) [16, 17]. Each SNP only has a small effect size and explains a small fraction of heritability. In order to account for this genetic nature, the polygenic risk score (PRS) was used to aggregate the effects of multiple SNPs from GWAS to capture the associations of genetic variants with PC and IBD. This approach has been successfully used to predict the risk of Alzheimer’s disease [18] and pan-cancer diseases [19].

**Fig. 1:**
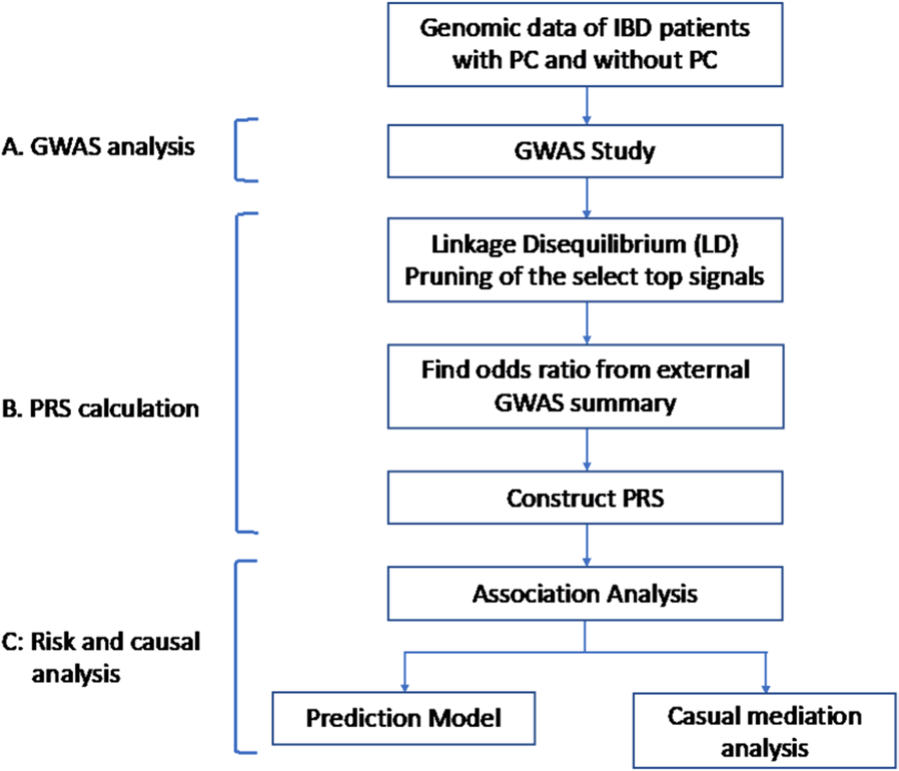
Flowchart of the overall analysis. The following analysis was conducted on the 240 persons with IBD: **A** GWAS analysis: risk SNPs selected at three P-value thresholds: 5 × 10^–2^, 5 × 10^–3^, and 5 × 10^–4^; **B** constructed PRS: two external GWAS summary: the IBD GWAS and the Autism GWAS (sensitivity analysis); **C** risk and causal analysis

Although there are many GWAS of IBD [16, 17], there are limited GWAS analyses of PC in IBD. Recently, we performed one of the first genome-wide copy number variation (CNV) analysis [20] and transcriptome-wide analysis of PC in IBD [21], which suggested that the expression level of the *RBPMS* gene has an association with PC status in persons with IBD [20, 21]. Gene expression has been considered as a mediator between genetic variants and disease phenotype [22]. Hence, we hypothesized that the genetic contribution to PC susceptibility among persons with IBD could be mediated by the expression level of *RBPMS*. In this study, we focused on persons with IBD of European ancestry. We first investigated the genetic contribution to the development of PC in IBD by constructing the genome-wide PRSs for each individual with IBD and examining its associations with the PC status in those persons. With a valid PRS demonstrated, we further showed that PRS can be used to predict the risk of PC. Finally, the causal mediation analyses were conducted to explore whether the expression level of the *RBPMS* gene is a mediator between the PRS and PC status in IBD (Fig. 1).

## Methods

### Study cohort

Samples for this study were collected from the Manitoba IBD Cohort Study which was initiated in 2002 [22]. The genetic profiles of the same samples were used for the previous CNV and transcriptome-wide analysis [20, 21]. The Manitoba IBD Cohort Study enrolled participants from the University of Manitoba IBD Research Registry. There were 3192 participants in the Research Registry database. The 606 of them who satisfied the following selection criteria were eligible for the study: (1) at least 18 years of age; (2) diagnosed with IBD within the previous 7 years. Among the 606 individuals, 388 provided baseline. Blood samples from 269 participants were available for genotyping. All participants in the Manitoba IBD Cohort Study were followed by annual in-person interviews and semi-annual mail surveys for 2 years. The IBD was diagnosed and phenotyped based on a chart review. The psychiatric disorders (depression and anxiety) were assessed through a structured psychiatric interview, the Comprehensive International Diagnostic Interview (CIDI) [23, 24] within 2 years of study enrolment. The CIDI is based on diagnostic criteria from the Diagnostic and Statistical Manual of Mental Disorders (DSM-IV) published by the American Psychiatric Association [25]. This study was approved by the University of Manitoba Health Research Ethics Board and all participants provided informed consent.

### Microarray genotyping and quality control

#### Genotype data

Genotyping was performed by The Centre for Applied Genomics at the Hospital for Sick Children on DNA samples extracted from blood using the Illumina Human Omin2.5M-8 platform (San Diego, CA, USA). This produced the genotype data for 2,372,784 SNPs for each individual. The quantity control (QC) was performed by PLINK version 1.9 [26] as described in the previous papers [20, 21]. In short, SNPs with missing rate $\ge$ 0.05, minor allele frequency < 0.05, and p-value < 5 × 10^–5^ for Hardy–Weinberg equilibrium were removed. There were 1,267,826 SNPs that passed genotype data QC and were included in the downstream analysis.

Twenty-six samples were removed by sample QC that was conducted by a previous study [20] due to insufficient call rate, genotyping errors, non-European ancestry (compared to The 1000 Genomes Project phase 3 data [27]), sample relatedness, or the inconsistency between self-reported and genotype measured sex. There were 240 participants (IBD with PC = 94 and IBD without PC = 146) left for the downstream analysis. The QC procedure ensured there was no bias produced by sample batch effect or genotyping quality.

#### Estimation of population structure

The multidimensional scaling (MDS) was used to identify the population structure of the samples included in the analysis. It was conducted based on the pairwise identity-by-state distance which was generated using SNPs that passed QC and with linkage disequilibrium (LD) below R^2^ = 0.2. The top five principal components were extracted and included in the GWAS analysis. The analysis was conducted by the PLINK v1.9 toolset [26].

#### Imputed expression levels of *RBPMS* gene

Transcriptome-wide association study of PC in IBD was performed on the same 240 samples [21]. We identified the *RBPMS* gene in skeletal muscle as a top signal with genetically regulated expression level associated (P-value = 5 × 10^–5^) with PC in persons with IBD of European ancestry. The expression level of *RBPMS* were imputed using PrediXcan [28]. PrediXcan predicts the gene expression data from an individual’s genetic profile based on a reference transcriptome database GTEx [29].

### Statistical analysis

#### Phenotypic association analyses

Exploratory analysis was performed to examine differences in the demographic characteristics between persons with IBD with PC and those without PC, including sex, age, disease subtypes, and marital status. The frequencies of each covariate were measured and compared between persons with IBD with PC and those without PC by chi-square tests. Statistical analyses were conducted in R version 3.5.1 [30].

#### Genome-wide association analysis

The GWAS analysis for PC status among persons with IBD of European ancestry was conducted on SNPs that passed QC using PLINK version 1.9 [26]. For each SNP, the logistic model with an additive genetic model was applied. Potential confounders, including population stratifications measured by the first five MDS components estimated based on genotype data that passed QC (see “Methods” in the previous section), age, sex, marital status, and disease subtypes were adjusted in the model.

1 $$\begin{aligned} logit \left( {PC\;status\;in\;IBD} \right) & \sim SNP + MDS1 + MDS2 + MDS3 + MDS4 \\ & \quad + MDS5 + sex + age + marital\;status + disease\;subtypes. \\ \end{aligned}$$

MDS is a statistical method which was used to identify the ethnicity outliers by comparing the SNP population frequencies in persons with IBD to the SNP frequencies in the reference populations. It was estimated in QC procedures. The odds ratio (OR) and P-values were obtained to assess the association between the allele counts and the risk of PC in IBD. The risk allele is the allele associated with greater disease risk and it was subsequently identified by comparing the ORs. The risk allele is the minor allele if the OR is > 1 and it is the major allele if the OR is < 1.

#### PRS association analysis of PC status in persons with IBD

PRS is an overall measure of an individual’s genetic liability to develop disease [31]. It is calculated as a weighted sum of the number of risk variants as shown by the Eq. (2)

2 $$PRS = \mathop \sum \limits_{i = 1}^{n} w_{i} x_{i} ,$$

where $x_{i}$ is the risk allele counts of each SNP from our study cohort, $w_{i}$ is the weight, which is the SNP association log odds ratio from external GWAS studies, and $n$ is the number of risk SNPs we selected. The risk SNPs were selected at three different P-value association thresholds: 5 × 10^–2^, 5 × 10^–3^ and 5 × 10^–4^. This approach can be considered as a parsimonious variable selection method, conducting forward selection based on P-values since the “optimal” P-value threshold is unknown [32]. For the selected risk SNPs, the pruning was performed to account for the independent associations from the GWAS study (R^2^ = 0.3, window size = 250 kB). Since there is no IBD/PC related GWAS study available, the external IBD GWAS [17] was used to compute the genome-wide PRSs for PC status in IBD in our cohort. The rationale for choosing the external IBD GWAS is that the phenotype is highly related to our target population. It contains 12,882 cases and 26,715 controls with only European ancestry (SNP = 11,555,662). In order to match the risk SNPs between our Manitoba IBD Cohort Study participants and the external dataset, the SNPs with mismatched alleles were excluded. The estimated PRS were further standardized for the comparison purpose. The association between standardized PRS and PC in IBD was then measured by logistic regression with and without adjusting the sex, age, marital status and disease subtypes (3, 4).

3 $$logit\;(PC\;status) \sim PRS,$$

4 $$logit\;(PC\;status) \sim PRS + sex + age + marital\;status + disease\;subtypes.$$

The log OR, confidence interval (CI), P-value, and McFadden pseudo-R^2^ were reported. The analysis was conducted in R [30].

#### Application of PRS to predict PC status in persons with IBD

Two machine learning methods, logistic regression and support vector machine (SVM) with a linear kernel, were used to construct models for predicting PC status in IBD using PRS and other demographic covariates. Specifically, sex, age, marital status, and disease subtypes. Predictive performance was measured by the receiver operator (ROC) curve and area under the curve (AUC) of the ROC calculated based on the tenfold cross-validation.

#### Causal mediation analysis

Regression-based mediation analyses were performed to examine whether the expression level of the *RBPMS* gene is mediated by the effect of PRS on PC status in persons with IBD of European ancestry. The analysis is based on the counterfactual framework of causal inference [33]. In the scenario of an absent the mediator, the research interest was the causal effect of exposure on the outcome. However, with the presence of the mediator, the total effect is no longer only directly coming from the exposure, but rather it can be decomposed into two parts: direct and indirect effect**.** The direct effect is the effect of exposure (estimated standardized PRS) on the outcome (PC status in IBD) with an absent the mediator (expression level of *RBPMS* gene). The indirect effect is the effect of PRS on the PC status in persons with IBD through the expression level of the *RBPMS* gene. The direct effect (${\uptheta }_{1}$) of the PRS on the PC status in IBD was measured by a logistic regression model, where the expression level of *RBPMS* was the mediator and sex, age, marital status, and disease subtypes were adjusted (5).

5 $$\begin{aligned} logit\left( {PC\;status} \right) & = \theta_{0} + \theta_{1} *PRS + \theta_{2} *RBPMS + sex + age \\ & \quad + marital\;status + disease\;subtypes \\ \end{aligned}$$

To estimate the indirect effect, model (6) was estimated by a linear regression model which treated the mediator (expression level of *RBPMS*) as the outcome and PRS as the exposure. Then the indirect effect is measured by $\emptyset_{1} *\theta_{2} ,$ where $\theta_{2}$ is the coefficient of *RBPMS* in Eq. (5) and $\emptyset_{1}$ is the coefficient of PRS in Eq. (6).

6 $$RBPMS = \emptyset_{0} + \emptyset_{1} *PRS + sex + age + marital\;status + disease\;subtypes.$$

The degree of mediation is measured by the proportion mediated, which is the ratio of the estimated indirect effect and the total effect (7).

7 $$\% \,{\text{mediated}} = \frac{{\emptyset_{1} * \theta_{2} }}{{\emptyset_{1} * \theta_{2} + \theta_{1} }}.$$

The confidence interval of the direct and indirect effect was computed based on the bootstrap of 1000 replications.

### Sensitivity analysis

In order to explore whether using different external datasets as weight in the PRS calculation would lead to a consistent result, we conducted the same set of analyses on autism spectrum disorder GWAS (autism GWAS) [34]. The rationale for choosing autism GWAS is that autism is one type of psychiatric disease. The dataset contains 18,381 cases and 27,969 controls for the population with European ancestry (SNP = 9,112,387).

## Results

### Demographic analysis

Demographic characteristics of PC group and those without PC were summarized in Table 1. Among the patients without PC, 49% were male, 58% were between 17 and 40 years of age, 47% had Crohn’s disease and 73% were married or had common-law relationships. The group with PC included fewer males (35%, P-value = 0.054), 64% were between 17 and 40 years of age at diagnosis, 53% had Crohn’s disease and 70% were married or in common-law relationships. There was no significant enrichment of PC in any of listed covariates, but we included all of them as potential confounders in the following analyses.

**Table 1:** Demographic characteristics of persons with IBD by PC status

N = 240	IBD without PC (N = 146)	IBD with PC (N = 94)	P-value^a^
Sex			0.054
Male	71 (48.6%^b^)	33 (35.1%)	
Female	75 (51.4%)	61 (64.9%)	
Age at diagnosis			0.4564
16 years and under	9 (6.16%)	3 (3.2%)	
17 to 40 years	84 (57.5%)	60 (63.8%)	
Over 40 years	53 (36.3%)	31 (33.0%)	
Disease subtype			0.444
Crohn’s disease	69 (47.3%)	50 (53.2%)	
Ulcerative colitis	77 (52.7%)	44 (46.8%)	
Marital status			
Married/common-law	106 (72.6%)	66 (70.2%)	0.799
Single/divorced/widowed	40 (27.4%)	28 (29.8%)	

^a^P-values are based on Pearson’s chi-squared test for categorical measures

^b^Among the persons with IBD without PC, 48.6% of them are male

### Genome-wide association analysis

GWAS on PC status adjusted by gender, age, marital status, disease subtypes, and the top 5 MDS components was visualized by Manhattan plot and QQ plot which are presented in Additional file 1: Fig. S1. There were no SNPs significantly associated with PC in IBD at the genome-wide threshold of (5 × 10^–8^). Although the power was limited by the sample size, which is shown in the QQ Plot, there were still 37 SNPs that had signals at the suggestive level (5 × 10^–5^). These 37 SNPs with suggestive significance were located at multiple chromosomes, which indicates the polygenic characteristic for this phenotype.

### PRS association analysis of PC status in persons with IBD

**Fig. 2:**
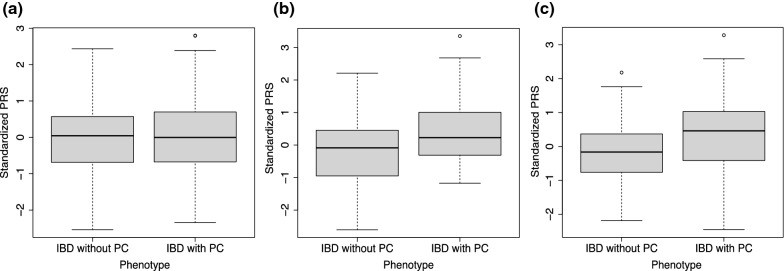
Boxplot of estimated PRS based on the IBD GWAS. **a** PRS for the European IBD GWAS at P-value threshold of 5 × 10^–2^. **b** PRS for the European IBD GWAS at P-value threshold of 5 × 10^–3^. **c** PRS for the European IBD GWAS at P-value threshold of 5 × 10^–4^

The number of risk SNPs identified at multiple P-value thresholds (5 × 10^–2^, 5 × 10^–3^ and 5 × 10^–4^) were 63,590, 5501 and 442, respectively, as shown in Table 2. The LD pruning was conducted on these risk SNPs in order to remove the correlated SNPs, leaving 21,748, 1104 and 72 SNPs among the three P-value thresholds respectively. The remaining SNPs were further matched with the IBD external GWAS dataset, with 9620, 471 and 33 SNPs matched at the three thresholds. For the 33 SNPs, all individuals included carried at least one risk allele. The top gene ontology functions enriched in the genes associated with these risk SNPs (Additional file 1: Table S3) are pyrimidine ribonucleotide metabolic process (GO:0009218), toll-like receptor 2 signaling pathway (GO:0034134) and protein localization to axon (GO:0099612). All of them have enriched P-value = 0.008 (adjusted P-value = 0.11). The details of the number of SNPs at risk after each step is shown in Table 2 and Additional file 1: Fig. S2. The distributions of the standardized PRS estimated based on IBD GWAS at different P-value thresholds are shown in Fig. 2. Persons with IBD with PC had slightly higher averaged standardized PRS than the group that did not have PC at 5 × 10^–3^ and 5 × 10^–4^ P-value thresholds. There is no significant difference at 5 × 10^–2^ P-value threshold. The results of PRS association between PC status in persons with IBD and the estimated PRS based on external IBD GWAS are presented in Table 3. The PRS had a significantly positive association with PC status at 5 × 10^–3^ and 5 × 10^–4^ P-value thresholds. The strongest and most significant effect was at the P-value threshold of 5*10^–3^ (OR = 1.9 and P-value = 1.9 × 10^–5^). The association was insignificant at the threshold of 5 × 10^–2^ (OR = 1.1 and P-value = 0.617). After adjusting for the covariates (sex, age, marital status, and disease subtypes), the association persisted and the threshold of 5 × 10^–3^ continues to have the strongest association with OR = 2.0 and P-value = 1.5 × 10^–5^.

**Table 2:** Number of SNPs identified from GWAS at different thresholds

	P-value threshold	Number of significant SNPs identified from GWAS under the threshold	Number of SNPs after LD pruning	Number of SNPs mapped to external IBD GWAS and used in the PRS calculation
1	5 × 10^–2^	63,590	21,748	9620
2	5 × 10^–3^	5501	1104	471
3	5 × 10^–4^	442	72	33

**Table 3:** Association analyses between PC status in persons with IBD and PRS estimated based on the IBD GWAS

	P-value threshold	Log OR	SE	P-value	R^2a^
PC^b^ ~ PRS	5 × 10^–2^	0.07	0.13	6.17E−01	0.001
	5 × 10^–3^	0.65	0.15	1.86E−05***	0.065
	5 × 10^–4^	0.61	0.15	3.64E−05***	0.059
PC ~ PRS + sex + age + marital status + disease subtypes	5 × 10^–2^	0.06	0.14	6.41E−01	0.023
	5 × 10^–3^	0.69	0.16	1.46E−05***	0.090
	5 × 10^–4^	0.67	0.15	1.50E−05***	0.089

^a^R^2^ is the McFadden’s pseudo R^2^

^b^PC stands for PC status in persons with IBD

***P-value ≤ 0.001

### Polygenetic risk analysis of PC status

**Fig. 3:**
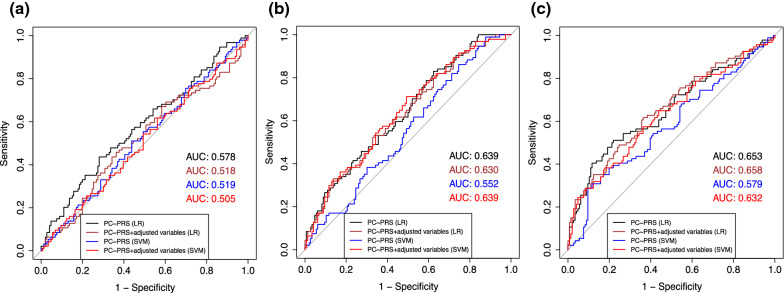
Receiver operator curves for PC status prediction at different P-value threshold estimated based on the IBD GWAS. **a** ROC for the European IBD GWAS at P-value threshold of 5 × 10^–2^. **b** ROC for the European IBD GWAS at P-value threshold of 5 × 10^–3^. **c** ROC for the European IBD GWAS at P-value threshold of 5 × 10^–4^

With the positive association between PRS and PC status demonstrated, we further examined the power of the PRS to predict PC status in persons with IBD using machine learning methods. The results are presented in Fig. 3. The threshold of 5 × 10^–4^ had the best prediction power among all three thresholds (AUC = 0.58–0.66). The logistic regression models with and without adjusting for covariates performed better than the SVM models.

### Causal mediation analysis

**Fig. 4:**
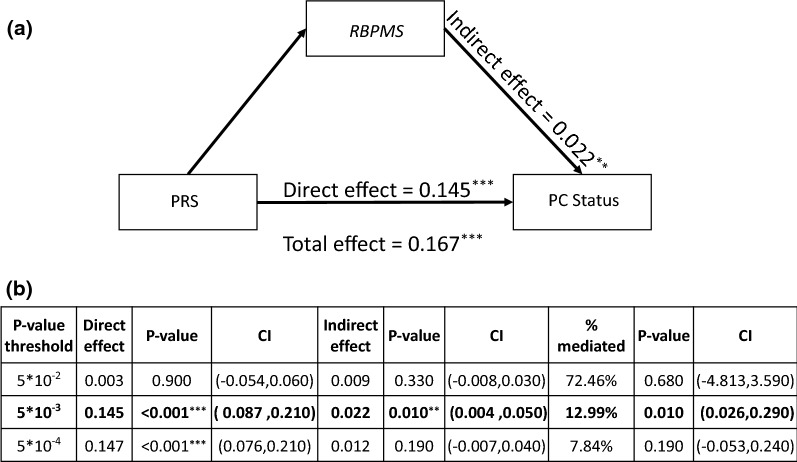
Casual mediation model. **a** Causal diagram between PRS estimated based on IBD GWAS, expression of *RBPMS* gene and PC status in IBD. **b** Causal mediation model result. *P-value ≤ 0.05; **P-value ≤ 0.01; ***P-value ≤ 0.001

In order to understand how different PRS will result in different PC status in persons with IBD of European ancestry, we further examined whether the association between PRS and PC status was mediated by the expression level of the *RBPMS* gene. We believe that different PRS will lead to the distinctive expression levels of the *RBPMS* gene and further result in different PC status. The diagram of the causal mediation models at the threshold of 5 × 10^–3^ are presented in Fig. 4a, and the result at all P-value thresholds are presented in Fig. 4b. For the PRS based on IBD GWAS, the results followed the same pattern as the previous section, with the P-value thresholds of 5 × 10^–3^ and 5 × 10^–4^ both having a strong significant direct effect. The P-value threshold of 5 × 10^–3^ also had a significant indirect effect. The estimated direct and indirect effects were 0.15 (95% CI 0.09, 0.21) and 0.022 (95% CI 0.004, 0.05), respectively. Around 13% of the total effect was mediated by the expression of the *RBPMS* gene.

### Sensitivity analyses

PRS based on the autism GWAS also resulted in a significant association with PC status in persons with IBD which verifies the robustness of our previous finding. The details of the results are shown in the Additional file 1. The association analysis between PRS and PC status in IBD was also conducted at three P-value thresholds (5 × 10^–2^, 5 × 10^–3^ and 5 × 10^–4^). The details of the number of SNPs at risk after mapping with external autism GWAS are shown in Additional file 1: Table S1. Over 96% of the identified SNPs were overlapped with the SNPs identified from the external IBD GWAS. The top gene ontology functions enriched in the genes associated with these risk SNPs at P-value threshold of 5 × 10^–4^ in the external autism GWAS (Additional file 1: Table S3) are also pyrimidine ribonucleotide metabolic process (GO:0009218), toll-like receptor 2 signaling pathway (GO:0034134) and protein localization to axon (GO:0099612). All of them have enriched P-value = 0.007 (adjusted P-value = 0.10). The estimated PRS had significant associations with PC status in IBD among all thresholds of P-values with the exclusion of the threshold of 5 × 10^–4^ (Additional file 1: Table S2). The estimated PRS had a similar prediction power compared to the PRS estimated based on IBD GWAS (Additional file 1: Figs. S3 and S4). In the causal mediation analysis, the direct effects were significant at both the thresholds of 5 × 10^–2^ and 5 × 10^–3^ and the indirect effects were not significant at any thresholds (at the threshold of 5 × 10^–2^: direct effect = 0.14, P-value < 0.001, 95% CI 0.072, 0.210; at the threshold of 5 × 10^–3^: direct effect = 0.12, P-value < 0.001, 95% CI 0.049, 0.190) (Additional file 1: Fig. S5).

## Discussion

In this analysis, we examined the association between estimated PRS and the PC status in persons with IBD of European ancestry and assessed the predictive power of the estimated PRS. In addition, we constructed a causal mediation model and understand the role of the expression level of the *RBPMS* gene among the association between the PRS and the PC status in IBD.

Our results showed that the PRS was significantly associated with the PC status in IBD at P-value thresholds of 5 × 10^–3^ and 5 × 10^–4^ when PRS was estimated based on the IBD GWAS. The positive odds ratio indicated that a higher PRS will result in a higher chance of having PC among persons with IBD. Our sensitivity analysis, using the autism GWAS as the weight to estimate PRS, also confirmed this result. The PRS based on the autism GWAS also had a positive association among all P-value thresholds, excluding the threshold of 5 × 10^–4^. The negative and nonsignificant association at the threshold of 5 × 10^–4^ for PRS estimated based on the autism GWAS may have two explanations. The first potential reason could be that only a small number of risk SNPs were used in the PRS calculation. We had 72 risk SNPs in our Manitoba IBD Cohort Study after LD and only 31 of them were in common with autism GWAS study. These 31 SNPs were used in the PRS calculation. However, the PC status in IBD is a polygenic phenotype which reflects the influence of many SNPs of small effect. Therefore, 31 SNPs may only capture a fraction of the effect that explains the nonsignificant P-value and an opposite direction of effect we observed. Secondly, both the IBD GWAS and the autism GWAS study are not directly related to our phenotype, which may account for the difference in the results.

PRS can potentially be used to predict the risk of PC status in persons with IBD of European ancestry. The highest AUC among all the models was 0.66, which suggested that PRS had power to predict PC status in IBD to some extent. The non-ideal prediction result could be due to the PRS being estimated based on using weights from indirectly related GWAS. Clinically, PRS could potentially be used for patient screening. Persons with high PC risk could receive psychiatric interventions such as cognitive behavioural therapy (CBT), medical hypnosis, mindfulness mediation, or pharmacotherapy [35]. Some studies have shown that CBT could improve quality of life, coping skills, and medical adherence [9]; medical hypnosis and mindfulness meditation could help to reduce gastrointestinal symptoms in persons with IBD. Therefore, early psychiatric interventions on persons predicted to have high PC risk could reduce the impact of mental ill-health and help to improve the long-term outcome of the course of IBD.

In the causal mediation analysis, we found that a small proportion of the effect of PRS on PC status in persons with IBD was mediated through the expression level of the *RBPMS* gene. *RBPMS* is an RNA binding protein and genetic variants on the *RBPMS* gene have been shown to be associated with psychiatric outcomes. From a meta-analysis of bipolar disorder GWAS, Nurnberger et al. [36] identified the *RBPMS* gene as one of the 226 genes associated with bipolar disorder. The *RBPMS* gene is also associated with optic nerve hypoplasia, hordeolum, and retinal ischemia. Therefore, it is possible that SNPs affecting PC status partially overlap with the gene *RBPMS*. Other unidentified genes may exist to explain the rest of the genetic contributions.

The present study has several limitations. First, the external GWAS study related to PC status in IBD is not available for the PRS calculation. Although the genetic variants in PRS calculation was selected based on the direct association of our IBD PC phenotype, the weights were borrowed from the external GWAS studies with other indirectly related phenotypes. This may lead to a reduced performance of the prediction model of PC status in IBD. Second, the sample size of our study is relatively small. The GWAS for IBD with PC is currently poorly studied. Our work can be considered as one of the first studies aiming to examine the association between PRS and PC among persons with IBD of European ancestry and to identify the mediation role of the *RBPMS* gene. We hope that our promising results will promote other large-scale studies on the topic in the future. Thirdly, since the analyses were restricted to individuals with European ancestry, the generalizability of the findings to other ethnic populations may be limited.

## Conclusion

We found a significantly positive association between PRS and PC status among persons with IBD, and around 13% of the effect could be explained by the expression level of the *RBPMS* gene. We also demonstrated that PRS can be used to predict the risk of PC among persons with IBD which could further be used for PC risk screening. Earlier diagnosis and treatment of PC can lead to better outcomes regarding mental health and course of IBD.

## Supplementary Information

**Additional file 1: Figure S1.** GWAS P-value results. (a) Manhattan Plot of GWAS P-value results. **Figure S2.** Distributions of the number of risk alleles of the SNPs that constitutes PRS carried by the tested samples (N = 240). **Figure S3.** Boxplot of estimated PRS based on the Autism GWAS. **Figure S4.** Receiver operator curves for PC status prediction at different P-value threshold estimated based on the autism GWAS. **Figure S5.** Casual mediation model. **Table S1.** Number of SNPs identified from GWAS at different thresholds and the number of SNPs used in the PRS calculation based on the Autism GWAS*.* **Table S2.** Polygenic association analyses between PC status in persons with IBD and PRS estimated based on the Autism GWAS. **Table S3.** **A** Genes associated with the identified risk SNPs (33) at P-value threshold of 5 × 10^−4^ based on external IBD GWAS. **B** Genes associated with the identified risk SNPs (N = 31) at P-value threshold of 5 × 10^−4^ based on external Autism GWAS.

## Data Availability

The datasets used and/or analysed during the current study are available from the corresponding author on reasonable request.
