# Supplementary material for: Polygenic risk and causal inference of psychiatric comorbidity in inflammatory bowel disease among patients with European ancestry
Source: J Transl Med. 2022 Jan 27;20:43. doi: 10.1186/s12967-022-03242-9 (PMC8793227; doi:10.1186/s12967-022-03242-9)
Supplement: Supplementary file 1 — Additional file 1: Figure S1. GWAS P-value results. (a) Manhattan Plot of GWAS P-value results. Figure S2. Distributions of the number of risk alleles of the SNPs that constitutes PRS carried by the tested samples (N = 240). Figure S3. Boxplot of estimated PRS based on the Autism GWAS. Figure S4. Receiver operator curves for PC status prediction at different P-value threshold estimated based on the autism GWAS. Figure S5. Casual mediation model. Table S1. Number of SNPs identified from GWAS at different thresholds and the number of SNPs used in the PRS calculation based on the Autism GWAS. Table S2. Polygenic association analyses between PC status in persons with IBD and PRS estimated based on the Autism GWAS. Table S3. A Genes associated with the identified risk SNPs (33) at P-value threshold of 5 × 10^−4^ based on external IBD GWAS. B Genes associated with the identified risk SNPs (N = 31) at P-value threshold of 5 × 10^−4^ based on external Autism GWAS. [file 12967_2022_3242_MOESM1_ESM.docx]

**Polygenic risk and causal inference of psychiatric comorbidity in inflammatory bowel disease among patients with European ancestry**

Yao Li^1,2^, Charles N Bernstein^3^, Wei Xu^1,4,#^, Pingzhao Hu^1,2,5,#,*^,

Additional file 1

**Figure S1.** GWAS P-value results. (a) Manhattan Plot of GWAS P-value results. The yellow, pink and brown lines are at the threshold of -log(5*10^-2^), -log(5*10^-3^) and -log(5*10^-4^) respectively. The blue line is at the suggestive significant level (-log(5*10^-5^)) and the red line is at the genome-wide significant level (-log(5*10^-8^)). (b) QQ Plot of GWAS P-value results. The dots in black are observed P-value distribution and the line in red is the expected P-value distribution.

**Figure S2.** Distributions of the number of risk alleles of the SNPs that constitutes PRS carried by the tested samples (N=240). Upper: SNPs matched with the external IBD GWAS with p-value threshold of (a) 5*10^-2^ (n=9620); (b) 5*10^-3^(n=471); (c) 5*10^-4^ (n=33). For the n=33 SNPs selected by the 5*10^-4^ threshold, every individual carries at least one risk allele. Lower: SNPs matched with the autism GWAS with the p-value threshold of (a) 5*10^-2^ (n=9181); (b) 5*10^-3^(n=439); (c) 5*10^-4^ (n=31).

**Figure S3.** Boxplot of estimated PRS based on the Autism GWAS. (a) PRS for the European Autism GWAS at P-value threshold of 5*10^-2^. (b) PRS for the European Autism GWAS at P-value threshold of 5*10^-3^. (c) PRS for the European Autism GWAS at P-value threshold of 5*10^-4^.

**Figure S4.** Receiver operator curves for PC status prediction at different P-value threshold estimated based on the autism GWAS. (a) ROC for the European autism GWAS at P-value threshold of 5*10^-2^. (b) ROC for the European autism GWAS at P-value threshold of 5*10^-3^. (c) ROC for the European autism GWAS at P-value threshold of 5*10^-4^.

**

**Figure S5.** Casual mediation model. (a) Causal diagram between PRS estimated based on the autism GWAS, expression of *RBPMS* gene and PC status in IBD. (b) Causal mediation model result. ^*^P-value ≤ 0.05; ^**^P-value ≤ 0.01; ^***^P-value ≤ 0.001.

**Table S1.** Number of SNPs identified from GWAS at different thresholds and the number of SNPs used in the PRS calculation based on the Autism GWAS.

|  | **P-value threshold** | **Number of significant SNPs identified from GWAS under the threshold** | **Number of SNPs after LD pruning** | **Number of SNPs mapped to external Autism GWAS and used in the PRS calculation** | **Number of overlapped mapped SNPs between IBD GWAS and Autism GWAS** |
| --- | --- | --- | --- | --- | --- |
| **1** | **5*10^-2^** | 63590 | 21748 | 9181 | 9103 |
| **2** | **5*10^-3^** | 5501 | 1104 | 439 | 437 |
| **3** | **5*10^-4^** | 442 | 72 | 31 | 30 |

**Table S2.** Polygenic association analyses between PC status in persons with IBD and PRS estimated based on the Autism GWAS.

|  | **P-value threshold** | **Log OR** | **SE** | **P-value** | **R^2a^** |
| --- | --- | --- | --- | --- | --- |
| **PC ~ PRS** | 5*10^-2^ | 0.62 | 0.15 | 3.58E-05^***^ | 0.059 |
|  | **5*10^-3^** | **0.54** | **0.14** | **1.66E-04^***^** | **0.048** |
|  | 5*10^-4^ | -0.21 | 0.13 | 1.22E-01 | 0.008 |
| **PC ~ PRS + sex + age** | 5*10^-2^ | 0.66 | 0.16 | 2.52E-05^***^ | 0.085 |
|  | **5*10^-3^** | **0.58** | **0.15** | **1.11E-04^***^** | **0.074** |
|  | 5*10^-4^ | -0.23 | 0.14 | 9.60E-02 | 0.031 |

^a^R^2^ is the McFadden's pseudo R^2^

^b^PC stands for PC status in persons with IBD

^***^P-value ≤ 0.001.

| **Table S3A:** Genes associated with the identified risk SNPs (33) at P-value threshold of 5*10^-4 based on external IBD GWAS | | | | |  |
| --- | --- | --- | --- | --- | --- |
| **Chromosome** | **Postion** | **Genetic variant** | **Corresponding gene** | **Functional annotation** |  |
|  |  |  |  |  |  |
| 1 | 17831661 | [rs7545559](https://pubs.broadinstitute.org/mammals/haploreg/detail_v4.1.php?query=&id=rs7545559) | 4.6kb 3' of ACTL8 |  |  |
| 1 | 17833597 | [rs11203472](https://pubs.broadinstitute.org/mammals/haploreg/detail_v4.1.php?query=&id=rs11203472) | 6.5kb 3' of ACTL8 |  |  |
| 1 | 41675581 | [rs17364300](https://pubs.broadinstitute.org/mammals/haploreg/detail_v4.1.php?query=&id=rs17364300) | HIVEP3 | intronic |  |
| 3 | 24770691 | [rs76428666](https://pubs.broadinstitute.org/mammals/haploreg/detail_v4.1.php?query=&id=rs76428666) | AC133680.1 |  |  |
| 6 | 36009409 | [rs6906520](https://pubs.broadinstitute.org/mammals/haploreg/detail_v4.1.php?query=&id=rs6906520) | SLC26A8 | intronic |  |
| 6 | 142743202 | [rs197488](https://pubs.broadinstitute.org/mammals/haploreg/detail_v4.1.php?query=&id=rs197488) | 5.2kb 5' of RP1-67K17.3 |  |  |
| 7 | 15039366 | [rs218066](https://pubs.broadinstitute.org/mammals/haploreg/detail_v4.1.php?query=&id=rs218066) | 27kb 5' of AC006458.3 |  |  |
| 7 | 93366440 | [rs10233804](https://pubs.broadinstitute.org/mammals/haploreg/detail_v4.1.php?query=&id=rs10233804) | 7.4kb 3' of CCDC132 |  |  |
| 7 | 105999714 | [rs2526978](https://pubs.broadinstitute.org/mammals/haploreg/detail_v4.1.php?query=&id=rs2526978) | CDHR3 | intronic |  |
| 7 | 148305489 | [rs6956705](https://pubs.broadinstitute.org/mammals/haploreg/detail_v4.1.php?query=&id=rs6956705) | CNTNAP2 | intronic |  |
| 7 | 154670149 | [rs28447185](https://pubs.broadinstitute.org/mammals/haploreg/detail_v4.1.php?query=&id=rs28447185) | DPP6 |  |  |
| 8 | 73197137 | [rs10093340](https://pubs.broadinstitute.org/mammals/haploreg/detail_v4.1.php?query=&id=rs10093340) | 73kb 5' of SBSPON |  |  |
| 8 | 118770216 | [rs10105083](https://pubs.broadinstitute.org/mammals/haploreg/detail_v4.1.php?query=&id=rs10105083) | 148kb 5' of SAMD12 |  |  |
| 9 | 4742001 | [rs1009447](https://pubs.broadinstitute.org/mammals/haploreg/detail_v4.1.php?query=&id=rs1009447) | AK3 | 5'-UTR |  |
| 9 | 76106739 | [rs7850358](https://pubs.broadinstitute.org/mammals/haploreg/detail_v4.1.php?query=&id=rs7850358) | PCSK5 | intronic |  |
| 9 | 112714360 | [rs10513168](https://pubs.broadinstitute.org/mammals/haploreg/detail_v4.1.php?query=&id=rs10513168) | INIP | intronic |  |
| 9 | 134604446 | [rs10115296](https://pubs.broadinstitute.org/mammals/haploreg/detail_v4.1.php?query=&id=rs10115296) | 19kb 3' of RP11-54A22.1 |  |  |
| 10 | 96668430 | [rs11188880](https://pubs.broadinstitute.org/mammals/haploreg/detail_v4.1.php?query=&id=rs11188880) | PIK3AP1 | intronic |  |
| 11 | 35528085 | [rs609664](https://pubs.broadinstitute.org/mammals/haploreg/detail_v4.1.php?query=&id=rs609664) | PAMR1 |  |  |
| 12 | 3937062 | [rs583801](https://pubs.broadinstitute.org/mammals/haploreg/detail_v4.1.php?query=&id=rs583801) | 27kb 3' of RP11-664D1.1 |  |  |
| 13 | 31353386 | [rs9531124](https://pubs.broadinstitute.org/mammals/haploreg/detail_v4.1.php?query=&id=rs9531124) | 21kb 3' of B3GALTL |  |  |
| 13 | 53088933 | [rs2806976](https://pubs.broadinstitute.org/mammals/haploreg/detail_v4.1.php?query=&id=rs2806976) | 37kb 3' of OLFM4 |  |  |
| 13 | 108853060 | [rs7991080](https://pubs.broadinstitute.org/mammals/haploreg/detail_v4.1.php?query=&id=rs7991080) | MYO16 | intronic |  |
| 14 | 99024042 | [rs72631664](https://pubs.broadinstitute.org/mammals/haploreg/detail_v4.1.php?query=&id=rs72631664) | 96kb 5' of RP11-1127D7.1 |  |  |
| 16 | 61561223 | [rs7202732](https://pubs.broadinstitute.org/mammals/haploreg/detail_v4.1.php?query=&id=rs7202732) | 86kb 3' of CDH8 |  |  |
| 16 | 86204249 | [rs13337791](https://pubs.broadinstitute.org/mammals/haploreg/detail_v4.1.php?query=&id=rs13337791) | 71kb 3' of Y_RNA |  |  |
| 17 | 8651988 | [rs7213982](https://pubs.broadinstitute.org/mammals/haploreg/detail_v4.1.php?query=&id=rs7213982) | 21kb 5' of MYH10 |  |  |
| 18 | 8982961 | [rs17495659](https://pubs.broadinstitute.org/mammals/haploreg/detail_v4.1.php?query=&id=rs17495659) | 120kb 5' of NDUFV2 |  |  |
| 19 | 35104318 | [rs12977462](https://pubs.broadinstitute.org/mammals/haploreg/detail_v4.1.php?query=&id=rs12977462) | AC020907.3 | intronic |  |
| 19 | 41028010 | [rs34855348](https://pubs.broadinstitute.org/mammals/haploreg/detail_v4.1.php?query=&id=rs34855348) | 299bp 5' of CYP2A7P1 |  |  |
| 19 | 46405711 | [rs35445518](https://pubs.broadinstitute.org/mammals/haploreg/detail_v4.1.php?query=&id=rs35445518) | 4.7kb 3' of CCDC8 |  |  |
| 21 | 20290022 | [rs9305949](https://pubs.broadinstitute.org/mammals/haploreg/detail_v4.1.php?query=&id=rs9305949) | 31kb 5' of AP001171.1 |  |  |
| 21 | 44756538 | [rs11701897](https://pubs.broadinstitute.org/mammals/haploreg/detail_v4.1.php?query=&id=rs11701897) | 13kb 3' of UBE2G2 |  |  |

| **Table S3B:** Genes associated with the identified risk SNPs (N=31) at P-value threshold of 5*10^-4 based on external Autism GWAS | | | | |  |
| --- | --- | --- | --- | --- | --- |
| **Chromosome** | **Postion** | **Genetic variant** | **Corresponding gene** | **Functional annotation** |  |
|  |  |  |  |  |  |
| 1 | 17831661 | [rs7545559](https://pubs.broadinstitute.org/mammals/haploreg/detail_v4.1.php?query=&id=rs7545559) | 4.6kb 3' of ACTL8 |  |  |
| 1 | 17833597 | [rs11203472](https://pubs.broadinstitute.org/mammals/haploreg/detail_v4.1.php?query=&id=rs11203472) | 6.5kb 3' of ACTL8 |  |  |
| 1 | 41675581 | [rs17364300](https://pubs.broadinstitute.org/mammals/haploreg/detail_v4.1.php?query=&id=rs17364300) | HIVEP3 | intronic |  |
| 3 | 24770691 | [rs76428666](https://pubs.broadinstitute.org/mammals/haploreg/detail_v4.1.php?query=&id=rs76428666) | AC133680.1 |  |  |
| 6 | 36009409 | [rs6906520](https://pubs.broadinstitute.org/mammals/haploreg/detail_v4.1.php?query=&id=rs6906520) | SLC26A8 | intronic |  |
| 6 | 142743202 | [rs197488](https://pubs.broadinstitute.org/mammals/haploreg/detail_v4.1.php?query=&id=rs197488) | 5.2kb 5' of RP1-67K17.3 |  |  |
| 7 | 15039366 | [rs218066](https://pubs.broadinstitute.org/mammals/haploreg/detail_v4.1.php?query=&id=rs218066) | 27kb 5' of AC006458.3 |  |  |
| 7 | 93366440 | [rs10233804](https://pubs.broadinstitute.org/mammals/haploreg/detail_v4.1.php?query=&id=rs10233804) | 7.4kb 3' of CCDC132 |  |  |
| 7 | 105999714 | [rs2526978](https://pubs.broadinstitute.org/mammals/haploreg/detail_v4.1.php?query=&id=rs2526978) | CDHR3 | intronic |  |
| 7 | 148305489 | [rs6956705](https://pubs.broadinstitute.org/mammals/haploreg/detail_v4.1.php?query=&id=rs6956705) | CNTNAP2 | intronic |  |
| 7 | 154670149 | [rs28447185](https://pubs.broadinstitute.org/mammals/haploreg/detail_v4.1.php?query=&id=rs28447185) | DPP6 |  |  |
| 8 | 73197137 | [rs10093340](https://pubs.broadinstitute.org/mammals/haploreg/detail_v4.1.php?query=&id=rs10093340) | 73kb 5' of SBSPON |  |  |
| 8 | 118770216 | [rs10105083](https://pubs.broadinstitute.org/mammals/haploreg/detail_v4.1.php?query=&id=rs10105083) | 148kb 5' of SAMD12 |  |  |
| 9 | 4742001 | [rs1009447](https://pubs.broadinstitute.org/mammals/haploreg/detail_v4.1.php?query=&id=rs1009447) | AK3 | 5'-UTR |  |
| 9 | 76106739 | [rs7850358](https://pubs.broadinstitute.org/mammals/haploreg/detail_v4.1.php?query=&id=rs7850358) | PCSK5 | intronic |  |
| 9 | 112714360 | [rs10513168](https://pubs.broadinstitute.org/mammals/haploreg/detail_v4.1.php?query=&id=rs10513168) | INIP | intronic |  |
| 10 | 96668430 | [rs11188880](https://pubs.broadinstitute.org/mammals/haploreg/detail_v4.1.php?query=&id=rs11188880) | PIK3AP1 | intronic |  |
| 12 | 3937062 | [rs583801](https://pubs.broadinstitute.org/mammals/haploreg/detail_v4.1.php?query=&id=rs583801) | 27kb 3' of RP11-664D1.1 |  |  |
| 12 | 129377643 | [rs7976435](https://pubs.broadinstitute.org/mammals/haploreg/detail_v4.1.php?query=&id=rs7976435) | TMEM132D | intronic |  |
| 13 | 31353386 | [rs9531124](https://pubs.broadinstitute.org/mammals/haploreg/detail_v4.1.php?query=&id=rs9531124) | 21kb 3' of B3GALTL |  |  |
| 13 | 53088933 | [rs2806976](https://pubs.broadinstitute.org/mammals/haploreg/detail_v4.1.php?query=&id=rs2806976) | 37kb 3' of OLFM4 |  |  |
| 13 | 108853060 | [rs7991080](https://pubs.broadinstitute.org/mammals/haploreg/detail_v4.1.php?query=&id=rs7991080) | MYO16 | intronic |  |
| 14 | 99024042 | [rs72631664](https://pubs.broadinstitute.org/mammals/haploreg/detail_v4.1.php?query=&id=rs72631664) | 96kb 5' of RP11-1127D7.1 |  |  |
| 16 | 61561223 | [rs7202732](https://pubs.broadinstitute.org/mammals/haploreg/detail_v4.1.php?query=&id=rs7202732) | 86kb 3' of CDH8 |  |  |
| 16 | 86204249 | [rs13337791](https://pubs.broadinstitute.org/mammals/haploreg/detail_v4.1.php?query=&id=rs13337791) | 71kb 3' of Y_RNA |  |  |
| 17 | 8651988 | [rs7213982](https://pubs.broadinstitute.org/mammals/haploreg/detail_v4.1.php?query=&id=rs7213982) | 21kb 5' of MYH10 |  |  |
| 18 | 8982961 | [rs17495659](https://pubs.broadinstitute.org/mammals/haploreg/detail_v4.1.php?query=&id=rs17495659) | 120kb 5' of NDUFV2 |  |  |
| 19 | 35104318 | [rs12977462](https://pubs.broadinstitute.org/mammals/haploreg/detail_v4.1.php?query=&id=rs12977462) | AC020907.3 | intronic |  |
| 19 | 41028010 | [rs34855348](https://pubs.broadinstitute.org/mammals/haploreg/detail_v4.1.php?query=&id=rs34855348) | 299bp 5' of CYP2A7P1 |  |  |
| 19 | 46405711 | [rs35445518](https://pubs.broadinstitute.org/mammals/haploreg/detail_v4.1.php?query=&id=rs35445518) | 4.7kb 3' of CCDC8 |  |  |
| 21 | 20290022 | [rs9305949](https://pubs.broadinstitute.org/mammals/haploreg/detail_v4.1.php?query=&id=rs9305949) | 31kb 5' of AP001171.1 |  |  |
